# A Novel Preparation Method for Camptothecin (CPT) Loaded Folic Acid Conjugated Dextran Tumor-Targeted Nanoparticles

**DOI:** 10.3390/ijms12074237

**Published:** 2011-06-28

**Authors:** Yuangang Zu, Dan Wang, Xiuhua Zhao, Ru Jiang, Qi Zhang, Dongmei Zhao, Yong Li, Baishi Zu, Zhiqiang Sun

**Affiliations:** Key Laboratory of Forest Plant Ecology, Northeast Forestry University, Ministry of Education, Harbin 150040, China; E-Mails: wangdan639@126.com (D.W.); 644677614@qq.com (R.J.); 835608075@qq.com (Q.Z.); 156828568@qq.com (D.Z.); 724506183@qq.com (Y.L.); zubaishi@163.com (B.Z.); zhiqiangshun@163.com (Z.S.)

**Keywords:** camptothecin, dextran, supercritical antisolvent, tumor-targeted, nanoparticle

## Abstract

In this study, folic-dextran-camptothecin (Fa-DEX-CPT) tumor-targeted nanoparticles were produced with a supercritical antisolvent (SAS) technique by using dimethyl sulfoxide (DMSO) as a solvent and carbon dioxide as an antisolvent. A factorial design was used to reveal the effect of various process parameters on the mean particle size (MPS) and morphology of the particles formed. Under the optimum operation conditions, Fa-DEX-CPT nanoparticles with a MPS of 182.21 nm were obtained. Drug encapsulation efficiency and loading efficiency were 62.13% and 36.12%, respectively. It was found that the concentrations of the camptothecin (CPT) and dextran solution had a major influence upon morphology and shape of the final product. In addition, the samples were characterized by Scanning electron microscopy (SEM), Fourier transform infrared spectroscopy (FT-IR), Differential scanning calorimetry (DSC) and X-ray diffraction (XRD) with the purpose of developing a suitable targeted drug delivery system for cancer chemotherapy.

## 1. Introduction

Camptothecin (CPT), a plant alkaloid, isolated from the stem wood of the oriental tree *Camptotheca acuminate* [1], has been reported to be a potent anticancer agent for a broad spectrum of cancers, such as gastrointestinal, ovarian, cervical, colorectal cancers and so on [2–8]. CPT acts by inhibiting the topoisomerase 1, an essential enzyme for the normal functioning of DNA [9,10], resulting in arresting the cell cycle in S phase and G_2_/M phase and inducing cell apoptosis [11,12]. However, poor water-solubility and the rapid hydrolysis of CPT-lactone to a ring-open carboxylate-form have limited CPT clinical development [13]. In addition, CPT can cause a number of toxic side effects to normal tissues [14–16]. In order to solve the aforementioned problems, several approaches have been studied, such as synthesis of new derivatives and pro-drugs. However, drug carriers have especially been developed. There are many studies about the lactone ring of CPT protected using drug delivery technologies like liposomes [17–19], polymers [20–22], dendritic molecules [23] and magnetic nanoparticles [24]. Furthermore, compared with CPT derivatives, CPT has higher activity [25]. Therefore, development of stable CPT has more prospects.

Dextran has highly water soluble ability and contains a large number of hydroxyl groups, which can be easily conjugated to drugs and proteins. In addition, conjugation with dextrans has resulted in prolongation of the effect, alteration of toxicity profile, and a reduction in the immunogenicity of drugs [26–27]. So in recent years, dextrans have been used in delivery of drugs, proteins and imaging agents [28–31]. In this paper, daxtran has been selected as the inclusion and releasing matrix to slow and long-term release of CPT. At the same time, the stability of the drug has been enhanced. Many targeting agents have been coupled with drug delivery systems to improve their site-specific targeting. Folic acid is a low-molecular-weight (441 Da) vitamin, the receptor for which is frequently overexpressed in human cancer cells, but is restricted in most normal tissues. Folate conjugates of radiopharmaceutical agents, chemotherapeutic agents, immunotherapeutic agents, and plasmids have all been delivered to cancer cells overexpressing the folate receptor. Therefore, we selected the folic acid as a target, assuming that the drug has more targets.

The supercritical antisolvent process (SAS) is a precipitation technology developed in recent years, which has been adapted for preparation of drug nanocrystals [32–37]. The advantages of the SAS process is that it is innocuous, low cost and pollution-free [38]. In this study, we prepared folic-dextran-camptothecin (Fa-DEX-CPT) tumor-targeted nanoparticles with a supercritical antisolvent (SAS) technique by using dimethyl sulfoxide (DMSO) as a solvent and carbon dioxide as an antisolvent. Moreover, the tumor-targeted nanoparticles were characterized by Scanning electron microscopy (SEM), Fourier transform infrared spectroscopy (FT-IR), Differential scanning calorimetry (DSC) and X-ray diffraction (XRD) with the purpose of developing a suitable targeted drug delivery system for cancer chemotherapy.

## 2. Materials and Methods

### 2.1. Materials

Dextran (DEX, Mw = 40,000) was purchased from Sinopharm Chemical Reagent Co. Ltd. (Shanghai City, China). Camptothecin (CPT, purity = 98.53%) was obtained from Sichuan Jiangyuan Natural Products Co. Ltd. (Sichuan, PR China). Folate (FA, purity ≥ 99.0%) was obtained from Sigma (Sigma Aldrich, St. Louis, MO, USA). Dimethyl sulfoxide (DMSO, purity ≥ 98.5%) was purchased from Sigma Aldrich. High purity CO_2_ (99.99% pure) was purchased from Liming Gas Company of Harbin (Heilongjiang, PR China).

### 2.2. Preparation of Fa-DEX-CPT Nanoparticles

#### 2.2.1. Preparation of Fa-DEX Powder

Dissolving 2 g of dextran in 60 mL of distilled water, followed by the addition of a desired amount of NaIO_4_ (5% molar equivalent). The mixture was stirred at 25 °C for 24 h, and then was lyophilized. Dissolving 10 mg of polyaldehyde in 8 mL of DMSO and adding 3.4 mg of folate. The reaction was performed for 24 h at 37 °C, and then an excess of acetic acid was added to gain precipitation. The precipitation was washed with ethanol and dried in the oven.

#### 2.2.2. SAS Process

The precipitation experiments were carried out in a SAS apparatus, which has been described elsewhere [39]. The Fa-DEX and CPT mixed solution was prepared by dissolving Fa-DEX and CPT in DMSO solution that acvcording to the concentration ratio. The supercritical CO_2_ is pre-heated in a heat exchanger and enters the precipitation chamber. Simultaneously, the mixture DMSO solution of Fa-DEX and CPT is injected into the SAS apparatus, also heated and fed to the 1000 mL precipitation chamber through a stainless steel nozzle. The flow rate of the mixture that leaves the precipitator is controlled by a valve located between the precipitation chamber and gas-liquid separation chamber. The liquid pump is stopped when injected the fixed quantity of DMSO solution. Continue to deliver supercritical CO_2_ for 30 min to wash the frit vessel from the residual content of liquid solubilized in the supercritical antisolvent. Finally, the samples of Fa-DEX-CPT nanoparticles were taken from the frit vessel for further characterization analysis.

#### 2.2.3. Factorial Design

Factorial design is a practical approach for studying multiple factors, with a minimum of experimental units [40,41]. Select for two-level factorial design to optimization of operating condition of Fa-DEX-CPT nanoparticles. Six parameters, (A) precipitation temperature (40~60 °C), (B) precipitation pressure (10~20 MPa), (C) nozzle diameter (200~300 μm), (D) drug solution flow rate (6.7~13.3 mL/min), (E) concentration of Fa-dextran solution (5~20 mg/mL) and (F) CPT solution (1~5 mg/mL) were chosen as variables, each one at two levels where 19 runs were employed which including a center point replicated 3 times. The center point is very important because it represents a set of experimental conditions at which three independent replicates were run. The variation between them reflects the variability of all design and it was used to estimate the standard deviation [42]. All experiments were performed in random order to avoid systematic errors. The data was analyzed using the Minitab 15 software (Minitab company, US).

### 2.3. Powder Characterization

#### 2.3.1. Scanning Electron Microscopy (SEM) Analysis

The morphology of particles was examined using SEM (Quanta 200, FEI). Samples were mounted on aluminum stubs using double sided carbon tape and sputter-coated with gold under an argon atmosphere.

#### 2.3.2. Mean Particle Size Analysis

The mean particle size (MPS) of the nanoparticles obtained was measured with a dynamic light scattering particle size analyzer (ZetaPALS, Brookhaven Instruments, USA). The sample powder was diluted to a concentration of 0.5% (wt/v) in deionised water and sonicated for 1 min before measurement. Mean particle size was the average of triplicate measurements for a single sample.

#### 2.3.3. Loading Efficiency (LE) and Drug Encapsulation Efficiency (EE)

Loading efficiency (LE) and drug encapsulation efficiency (EE) of the Fa-dextran-CPT nanoparticles were determined by a high performance liquid chromatograph (HPLC, Waters Corporation, Milford, MA, USA) at 25 °C with a reverse-phase Diamonsil C_18_ column (250 mm × 4.6 mm; Dikma Technologies, Beijing, China). Injected 10 μL samples into C_18_ column using 3:7 acetonitrile/ultraputre water as the mobile phase (flow rate of 1.0 mL/min). The detection wavelength was 254 nm. The experiments were performed in triplicate. Drug loading efficiency and drug encapsulation efficiency were calculated by formula (1) and (2), respectively.

(1) $$\text{LE}\%=\frac{\text{Weight of the drug in nanoparticles}}{\text{Weight of the nanoparticles}}\times 100\%$$

(2) $$\text{EE}\%=\frac{\text{Weight of the drug in nanoparticles}}{\text{Weight of the feeding drugs}}\times 100\%$$

#### 2.3.4. Determination of Folate Content Associated with the Dextran NPs

Weighed an amount of Fa-DEX-CPT nanopartilces accurately and dissolved in DMSO. Determined the absorbance at 281 nm and calculated the folate content by the linear regression equation obtained from standard curve. The content of the conjugated folate was the mean of triplicate experiments.

#### 2.3.5. Fourier-Transform Infrared Spectroscopy (FTIR) Analysis

FT-IR spectra were obtained by MAGNA-IR560 E.S.P (Nicolet, USA) and recorded in the wave number range of 400–4000 cm^−1^ at a resolution of 4 cm^−1^. Sample powder was dried at 80 °C for 24 h, then, mixed with KBr powder at 1%, pressed to obtain self-made sample disks.

#### 2.3.6. X-ray Diffraction (XRD) Analysis

XRD diffraction analyses were carried out in order to characterize crystalline products. Powder X-ray diffraction patterns of samples were obtained using power X-ray diffractometer (Philips, Xpert-Pro, The Netherlands) with Cu Ka_1_ radiation generated at 30 mA and 50 kV. The samples were run over the most informative range from 3° to 90° of 2θ.

#### 2.3.7. Differential Scanning Calorimeters (DSC) Analysis

Thermal analysis of the particles was studied by DSC (TA instruments, model DSC 204). Approximately 5 mg of particles were loaded onto standard aluminum pans. The samples were purged with pure dry nitrogen at a flow rate of 5 mL/min. The analysis was carried out at a temperature heating rate of 5 °C/min and a temperature range of 20–270 °C.

## 3. Results and Discussion

### 3.1. Effect of Operating Parameters on the Mean Particles Size of Fa-DEX-CPT

The experiment condition and the collected date for mean particle size of Fa-DEX-CPT nanoparticles obtained are shown in Table 1. It can be seen from the table that the maximum MPS of Fa-DEX-CPT nanoparticles was 736.74 nm, and the minimum was 78.99 nm.

**Figure 1:**
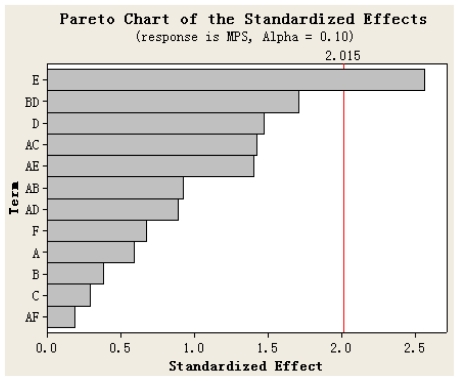
Standardized Pareto chart for mean particle size of Fa-DEX-CPT nanoparticles.

The Pareto chart (Figure 1) gives the relative importance of the individual and interaction effects. Student’s *t*-test was performed to determine whether the calculated effects were significantly different from zero and these values for each effect are shown in Pareto chart by horizontal columns [43]. For a 95% confidence level and sixteen degrees of freedom *t*-value is equal to 2.015. The vertical line in the chart indicates the minimum statistically significant effect magnitude for 95% confidence level. It can be seen from Figure 1 that the concentration of Fa-dextran solution (E) is the significant variable. At the same time, the influence to the MPS of Fa-DEX-CPT nanoparticles decreases in the order: E > D > F > A > B > C according to the standardized effect values.

### 3.2. Main Effect Analysis

**Figure 2:**
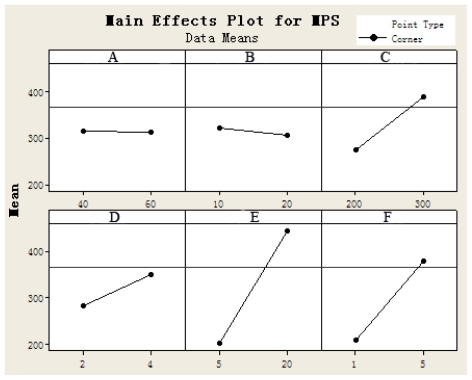
The main effects plot of parameters for mean particle size of Fa-DEX-CPT nanoparticles.

The main effects plot of parameters (Figure 2) shows the trends of all effects and it could be assumed from this plot that increase in precipitation pressure leads to decrease in mean particle size of Fa-DEX-CPT nanoparticles, but increase in other parameters results in formation of larger particles. Combine the results of the experiments, Fa-DEX-CPT nanoparticles with a MPS of 182.21 nm was obtained under optimum conditions of precipitation temperature of 40 °C, precipitation pressure of 20 MPa, drug solution flow rate of 7 mL/min and nozzle diameter of 200 μm, concentration of Fa-DEX solution of 5 mg/mL, concentration of CPT solution of 4 mg/mL by Minitab 15 software, respectively. Under these optimum conditions, drug encapsulation efficiency and loading efficiency were 62.13% and 36.12%, respectively.

### 3.3. Morphology of Fa-DEX-CPT Nanoparticles

**Figure 3:**
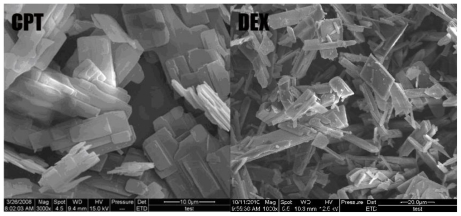
SEM images of unprocessed CPT and unprocessed dextran.

Figure 3 shows a SEM picture of unprocessed CPT and unprocessed dextran. It can be seen that unprocessed CPT particles are irregular lamelliform crystals, ranging in length from 1 to 80 μm, and the unprocessed dextran particles are flake crystals, ranging in length from 10 to 120 μm. CPT particles obtained by SAS process are spherical with a mean particle size of 245.5 ± 20.3 nm [39].

**Figure 4:**
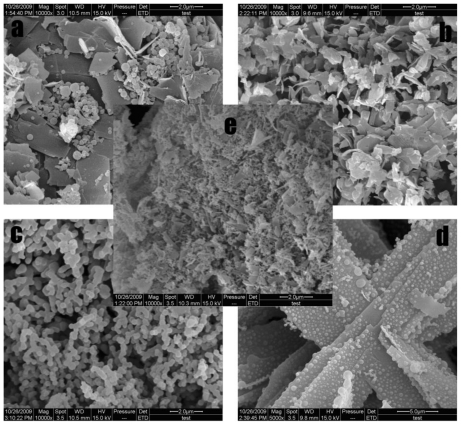
SEM images of Fa-DEX-CPT nanoparticles precipitated from DMSO under typical factorial experimental design conditions.

Fractional experiments showed that Fa-DEX-CPT has different morphologies and MPS under different operating conditions (Table 1). Figure 4 gives several typical morphologies of the samples obtained under different processing conditions. Figure 4a shows the sample is a mixture of spherical and flake microparticles. This fact can be attributed to the high concentration of dextran. It is obvious that the all particles present flake crystals (Figure 4b). As shown in Figure 4c, agglomerated spherical structures were obtained. Figure 4d shows an interesting result, *i.e.*, the formation of lamellar dextran with aggregated spheres CPT particles. Figure 4e shows a mixture of most beads and some flake crystals under the central point.

### 3.4. Surface Chemistry of Fa-DEX-CPT Nanoparticles

Folate had an absorption maximum at 281 nm and there was no apparent absorption peak of CPT. Using the linear regression equation obtained from standard curve: *y* = 0.0687*x* + 0.0136; *R^2^* = 0.9986, it was found that the folate content associated with dextran was up to 2.97% (wt).

**Figure 5:**
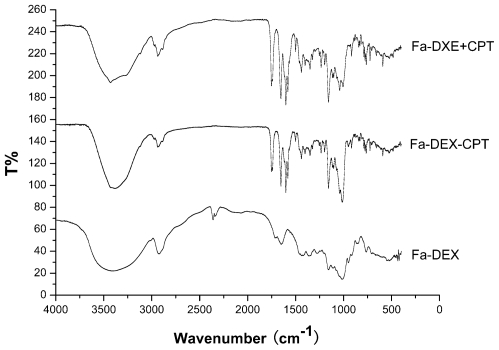
FTIR spectra of dextran, mixture of dextran and CPT, and Fa-DEX-CPT nanoparticles precipitated from DMSO under optimum conditions.

Figure 5 shows the FTIR results for raw dextran, mixture of dextran and CPT, and Fa-DEX-CPT nanoparticles precipitated from DMSO under optimum conditions. The FTIR spectra of raw dextran showed the presence of the following speaks: 3404 cm^−1^, 2931 cm^−1^, 1746 cm^−1^, 1652 cm^−1^ and 1161 cm^−1^. We can see from the FTIR spectra between mixture of dextran and CPT and Fa-DEX-CPT nanoparticles that no significant differences were shown. It can also be seen that the molecular structure of processed CPT has no change.

**Figure 6:**
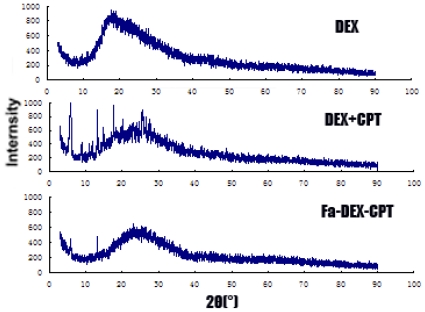
XRD spectra of dextran, mixture of dextran and CPT, and Fa-DEX-CPT nanoparticles precipitated from DMSO under optimum conditions.

XRD results for raw dextran, mixture of dextran and CPT, and Fa-DEX-CPT nanoparticles precipitated from DMSO under optimum conditions are reported in Figure 6. It can be clearly seen that the X-ray diffractogram of the three samples are generally similar. However, there were several distinct peaks in the X-ray diffractogram of the mixture of dextran and CPT at the diffraction angles of 2θ = 5.8°, 8.8°, 11.0°, 1.3°, 14.8°, 17.5°, 20.2° and 25.6°. Furthermore, in the X-ray diffractogram of the Fa-DEX-CPT nanoparticles, only several diffract peaks at 5.8°, 13.2° and 23.1° are present, which suggests that Fa-DEX-CPT nanoparticles obtained had low crystallinity after the SAS processing.

**Figure 7:**
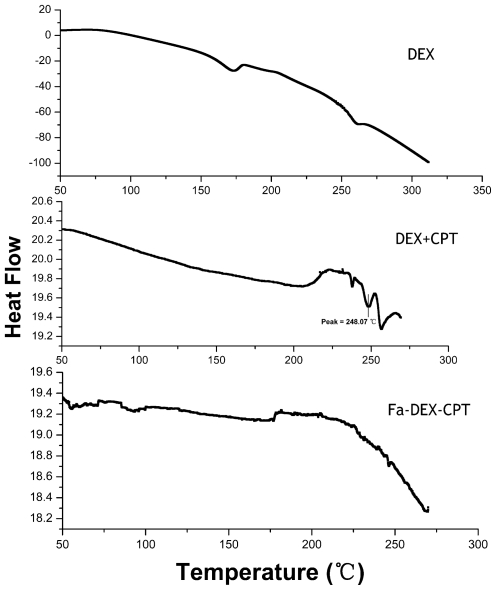
DSC spectra of dextran, mixture of dextran and CPT, and Fa-DEX-CPT nanoparticles precipitated from DMSO under optimum conditions.

The DSC thermograms of raw dextran, mixture of dextran and CPT, and Fa-DEX-CPT nanoparticles precipitated from DMSO under optimum conditions are shown in Figure 7. It can be seen that the mixture of dextran and CPT showed two endothermic melting peaks at 248.07 °C and 255.86 °C. However, raw dextran and Fa-DEX-CPT nanoparticles almost did not present any endothermic peak. This evidence confirms that the CPT in Fa-DEX-CPT nanoparticles has a lower crystallinity after SAS processing.

## 4. Conclusions

This study has shown that the SAS process can be successfully utilized to prepare Fa-DEX-CPT tumor-targeted nanoparticles. Compared with an unprocessed drug, Fa-DEX-CPT tumor-targeted nanoparticles obviously decreased in diameter. In addition, it has more specific target to tumor. Under the optimum operation conditions, Fa-DEX-CPT nanoparticles with an MPS of 182.21 nm were obtained. In particular, the mean particle size of Fa-DEX-CPT nanoparticles was strongly influenced by the drug concentration and the drug solution flow rate. Drug encapsulation efficiency and loading efficiency were 62.13% and 36.12% (common level is 12–22% loading efficiency [44]), respectively. Fa-DEX-CPT nanopartilces has different morphologies such as flake crystals, bead crystals, mixture of spherical and flake microparticles and MPS under different operating conditions. Compared with other methods for preparing tumor-targeted nanoparticles, the SAS process is uncomplicated to implement. Moreover, FTIR, XRD and DSC allowed the characterization of Fa-DEX-CPT nanoparticles. Physicochemical characterization results showed that the SAS process has not induced degradation of CPT, which suggests Fa-DEX-CPT nanoparticles have excellent potential in drug delivery systems for cancer chemotherapy.

## Figures and Tables

**Table 1:** Fractional factorial experimental design matrix and results.

Run	Sample No.	Dependent Variables						Result	

		A (°C)	B (MPa)	C (μm)	D (mL/min)	E (mg/mL)	F (mg/mL)	Mean Particle Size (nm)	Picture
1	12	40.00	10.00	200.00	6.7	5.00	1.00	160.26	
2	15	60.00	10.00	200.00	6.7	20.00	1.00	231.74	
3	13	40.00	20.00	200.00	6.7	20.00	5.00	451.62	
4	14	60.00	20.00	200.00	6.7	5.00	5.00	126.14	
5	2	40.00	10.00	300.00	6.7	20.00	5.00	676.3	
6	3	60.00	10.00	300.00	6.7	5.00	5.00	251.94	
7	4	40.00	20.00	300.00	6.7	5.00	1.00	78.99	
8	5	60.00	20.00	300.00	6.7	20.00	1.00	251.94	Figure 4a
9	9	40.00	10.00	200.00	13.3	5.00	5.00	402.19	
10	16	60.00	10.00	200.00	13.3	20.00	5.00	263.87	
11	11	40.00	20.00	200.00	13.3	20.00	1.00	755.69	
12	10	60.00	20.00	200.00	13.3	5.00	1.00	434.18	Figure 4c
13	6	40.00	10.00	300.00	13.3	20.00	1.00	307.79	
14	17	60.00	10.00	300.00	13.3	5.00	1.00	262.54	
15	8	40.00	20.00	300.00	13.3	5.00	5.00	126.23	Figure 4d
16	7	60.00	20.00	300.00	13.3	20.00	5.00	736.74	Figure 4b
17	1	50.00	15.00	300.00	10	12.50	3.00	482.6	Figure 4e
18	18	50.00	15.00	300.00	10	12.50	3.00	465.37	
19	19	50.00	15.00	300.00	10	12.50	3.00	479.58	
